# New Method of Attaching Gold Crowns to Natural Roots of Teeth

**Published:** 1879-01

**Authors:** 


*New Method of Attaching Gold Crowns to Natural Roots of Teeth.*—Dr. A. S. Richmond has recently visited Baltimore for the purpose of introducing Dr. C. M. Richmond's new method of forming and attaching artificial crowns of gold to natural roots without the use of adhesive gold foil. The crowns consist of twenty-two carat gold for molar and bicuspid roots, so shaped as to resemble those of the natural teeth and attached to the necks of the roots so firmly that they can be antagonized with teeth in the opposite jaw and withstand the force of mastication, and at the same time protect the roots from attacks of caries.

The artificial incisor and cuspid crowns differ from those of the bicuspids and molars in not being wholly composed of gold; an ordinary plate tooth being used in connection with the gold band which forms the bond of attachment with the natural root. Should this method prove as durable as the inventor claims for it, roots which are otherwise useless, may be rendered serviceable and sightly. Dr. Richmond occupied the greater part of two days in demonstrating this new method to the large class of the Baltimore College of Dental Surgery, the patients being students of the College.

He has also instructed a number of dental practitioners of Baltimore in this new method.

